# Supplementary material for: Green Optimization of Sesame Seed Oil Extraction via Pulsed Electric Field and Ultrasound Bath: Yield, Antioxidant Activity, Oxidative Stability, and Functional Food Potential
Source: Foods. 2025 Oct 26;14(21):3653. doi: 10.3390/foods14213653 (PMC12607709; doi:10.3390/foods14213653)
Supplement: Supplementary file 1 [file foods-14-03653-s001.zip › foods-3946684-supplementary.pdf]

foods

MDPI

Supplementary material

Green Optimization of Sesame Seed Oil Extraction via Pulsed Electric Field and Ultrasound Bath: Yield, Antioxidant Activity, Oxidative Stability, and Functional Food Potential

Vassilis Athanasiadis, Marianna Giannopoulou, Georgia Sarlami, Eleni Bozinou, Panagiotis Varagiannis and Stavros I. Lalas *

Department of Food Science and Nutrition, University of Thessaly, Terma N. Temponera Street, 43100 Karditsa, Greece; vaathanasiadis@uth.gr (V.A.); mgiannop@uth.gr (M.G.); gsarlami@uth.gr (G.S.); empozinou@uth.gr (E.B.); pvaragiannis@uth.gr (P.V.)

* Correspondence: slalas@uth.gr; Tel.: +30-24410-64783

Model Analysis

The polynomial regression Equations for Fat content (S1), DPPH (S2), CDs (S3), and CTs (S4) capture the relationships among the coded independent variables X_1 (energy power), X_2 (liquid-to-solid ratio), and X_3 (extraction time) through linear, quadratic, and interaction terms, enabling nuanced interpretation of their combined effects. Block-specific adjustments, expressed as Block[1] for PEF and Block[2] for UBAE, account for systematic differences between techniques by applying constant shifts and, where retained, factor-dependent slope changes. Across responses, X_2 emerges as a key driver, with positive main effects in the fat content and DPPH models and negative effects in CTs, while in CDs it is positive overall but modified by negative block-specific slopes under PEF. X_3 is generally positive for fat content, CDs, and CTs, but DPPH includes a negative linear time term alongside smaller quadratic curvature, indicating potential decline at long extractions. X_1 shows variable influence—slightly negative for fat content, strongly negative in the DPPH linear term but with a small positive quadratic, slightly negative for CDs, and slightly positive for CTs—reflecting technique-dependent roles. Interaction effects are response-specific: $X_2 \times X_3$ is negative in fat content and CDs but positive in DPPH, $X_1 \times X_3$ is negative in DPPH and CDs, and $X_1 \times X_2$ is small and positive only in DPPH and CTs. The asymmetric Block[1]/Block[2] corrections refine intercepts and selected slopes between techniques, underscoring the importance of modeling contextual variability to capture both global trends and block-level nuances.

$$Fat = 22.46 - 0.112X_1 + 1.596X_2 + 0.901X_3 - 0.0439X_2X_3 + \text{Block[1]}(-6.78 - 9.00 + 0.600X_2 - 6.49 + 0.324X_3) + \text{Block[2]}(+6.78 + 9.00 - 0.600X_2 + 6.49 - 0.324X_3) \quad \text{(S1)}$$

$$DPPH = 168.13 - 5.446X_1 + 3.447X_2 + 2.886X_3 + 0.0379X_1^2 - 0.1775X_2^2 - 0.0296X_3^2 + 0.0167X_1X_2 - 0.0352X_1X_3 + 0.0411X_2X_3 + \text{Block[1]}(3.626 + 3.891 - 0.2594X_2 + 6.939 - 0.0867X_1) + \text{Block[2]}(-3.626 - 3.891 + 0.2594X_2 - 6.939 + 0.0867X_1) \quad \text{(S2)}$$

$$CDs = 5.456 - 0.243X_1 + 0.904X_2 + 2.052X_3 + 0.004X_1^2 - 0.0183X_1X_3 - 0.0406X_2X_3 + \text{Block[1]}(8.73 - 0.109X_1 - 0.236X_2) + \text{Block[2]}(-8.73 + 0.109X_1 + 0.236X_2) \quad \text{(S3)}$$

$$CTs = -3.114 + 0.156X_1 - 0.743X_2 + 0.729X_3 - 0.00104X_1^2 + 0.0237X_2^2 - 0.0129X_3^2 + 0.00294X_1X_2 - 0.00147X_1X_3 - 0.00737X_2X_3 + \text{Block[1]}(-0.766) + \text{Block[2]}(+0.766) \quad \text{(S4)}$$

For fat yield, the UBAE block showed a higher baseline compared to PEF, consistent with cavitation-driven matrix disruption. For DPPH, the PEF block baseline was higher, reflecting better preservation of radical-scavenging

compounds under electroporation. For conjugated dienes (CDs), the PEF baseline was lower than UBAE, indicating reduced primary oxidation under electric field treatment. For conjugated trienes (CTs), UBAE at low energy/time combinations yielded lower baseline values, while PEF responses were more sensitive to energy–time interactions.

The regression and ANOVA results (Table S1) indicate that the fitted stepwise-BIC models explain a substantial proportion of the variability for all four responses, with (R^2) values of 0.850 for fat content, 0.982 for DPPH, 0.853 for CDs, and 0.871 for CTs. Fat content and DPPH in particular showed strong explanatory power with high adjusted (R^2) (0.745 and 0.937, respectively), underscoring the relevance of the retained predictors. For fat content, the liquid-to-solid ratio (X_2) has a consistent positive effect, complemented by block effects and, to a lesser extent, extraction time. In the DPPH model, prominent quadratic (X_1^2) and interaction ($X_1 \times X_3$) terms, alongside a positive block effect, highlight pronounced curvature and factor interplay. The CDs model retains several interaction terms (e.g., $X_1 \times X_3$, $X_2 \times X_3$) and X_1^2, indicating sensitivity to combined changes in energy and time, while CTs are influenced by X_2, $X_2 \times X_3$ and X_3^2. Significant blocking effects in multiple models confirm that extraction technique meaningfully shifts response baselines. Although some lack-of-fit tests were significant—particularly for fat content, DPPH, and CTs—the overall model structure remains informative, and the combination of high (R^2), acceptable RMSE, and coherent effect patterns demonstrates that the stepwise-selected second-order models provide a robust basis for interpreting and optimizing the extraction process. Although the models showed strong explanatory power, lack-of-fit was significant for Fat, DPPH, and CTs ($p < 0.05$), indicating residual structure not captured by the quadratic form; accordingly, inference is restricted to the experimental domain, and predictions outside the tested ranges should be treated as provisional and validated by confirmation runs.

Table S1. ANOVA results for the fitted quadratic polynomial models in the response surface methodology, showing the significance of main, quadratic, and interaction terms for fat content, DPPH activity, conjugated dienes (CDs), and conjugated trienes (CTs).

Factor	Fat	DPPH	CDs	CTs
Stepwise Regression				
Intercept	42.29*	8.757*	24.72*	5.267*
Block[1]	-6.78*	3.626*	-3.55*	-0.77*
X_1—energy power	-2.24	3.323*	0.611	0.086
X_2—liquid-to-solid ratio	3.591*	1.402	0.457	0.271*
X_3—extraction time	2.427	-4.99*	-0.22	-0.15
Block[1]X_1	-	-1.73	-2.18	-
X_1^2	-	15.17*	1.6	-0.42
Block[1]X_2	2.999	-1.3	-1.18	-
X_1X_2	-	1.67	-	0.294
X_2^2	-	-4.44	-	0.592
Block[1]X_3	3.243	-	-	-
X_1X_3	-	-7.04*	-3.66*	-0.29
X_2X_3	-2.19	2.055	-2.03*	-0.37*
X_3^2	-	-2.96	-	-1.29*
ANOVA				
F-value (model)	8.10	22.25	5.16	4.72
F-value (lack of fit)	97.97	215.37	9.87	38.44
p-Value (model)	0.0019*	0.0015*	0.0152*	0.0256*
p-Value (lack of fit)	0.0102*	0.0046	0.0949 ^{ns}	0.0255*
R^2	0.850	0.982	0.853	0.871
Adjusted R^2	0.745	0.937	0.688	0.686
RMSE	4.78	2.55	2.62	0.37
MR	42.29	14.80	25.96	4.40
CV	22.39	68.8	18.09	15.12
DF (total)	17	17	17	17

* Values marked with an asterisk were significant at the 95% probability level ($p < 0.05$). ns, non-significant; *F*-value, ratio comparing model variance with residual (error) variance; *p*-value, probability of observing the reported *F*-value under the null hypothesis; RMSE, root mean square error; MR, mean of response; CV, coefficient of variation; DF, degrees of freedom.

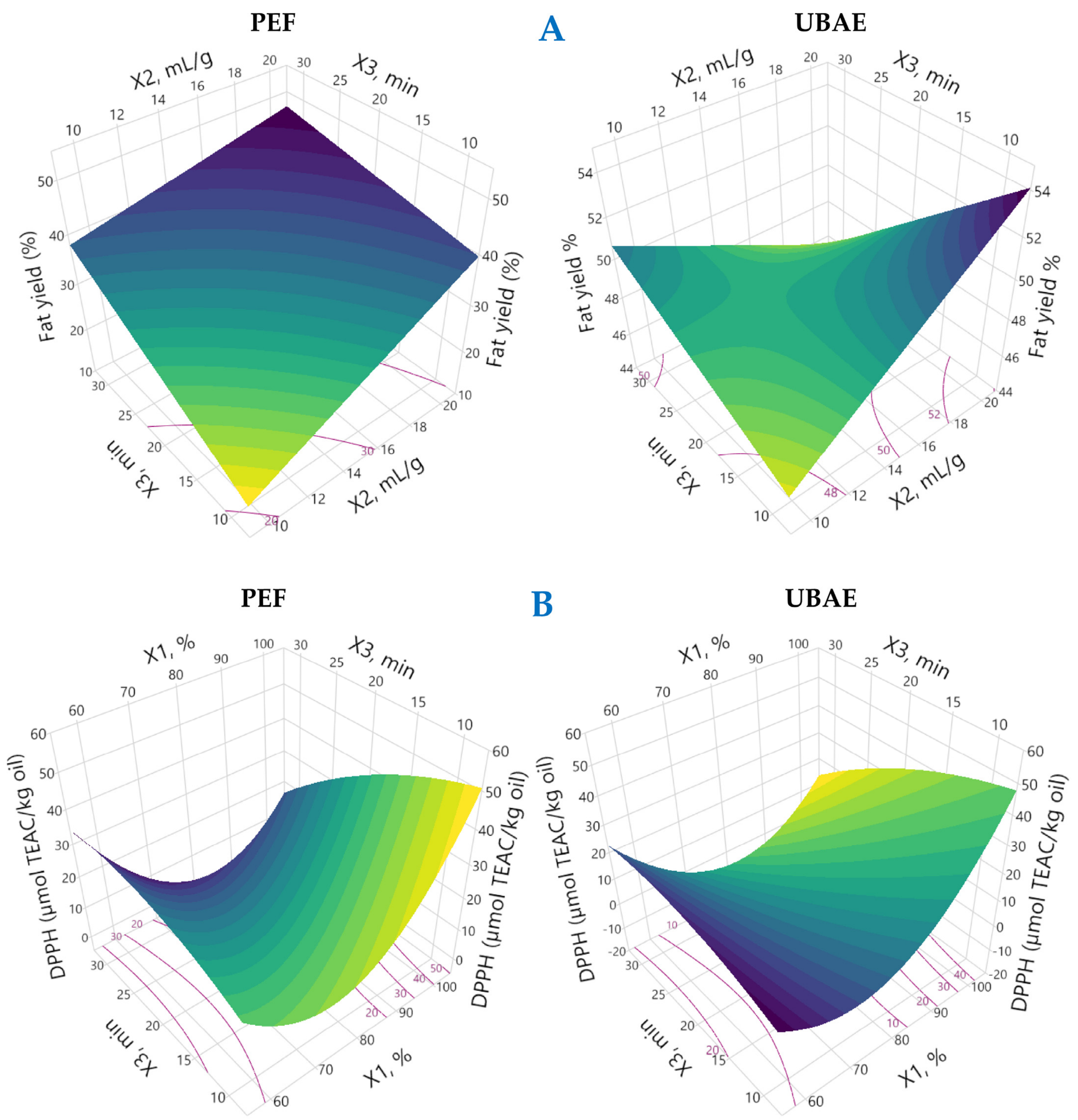

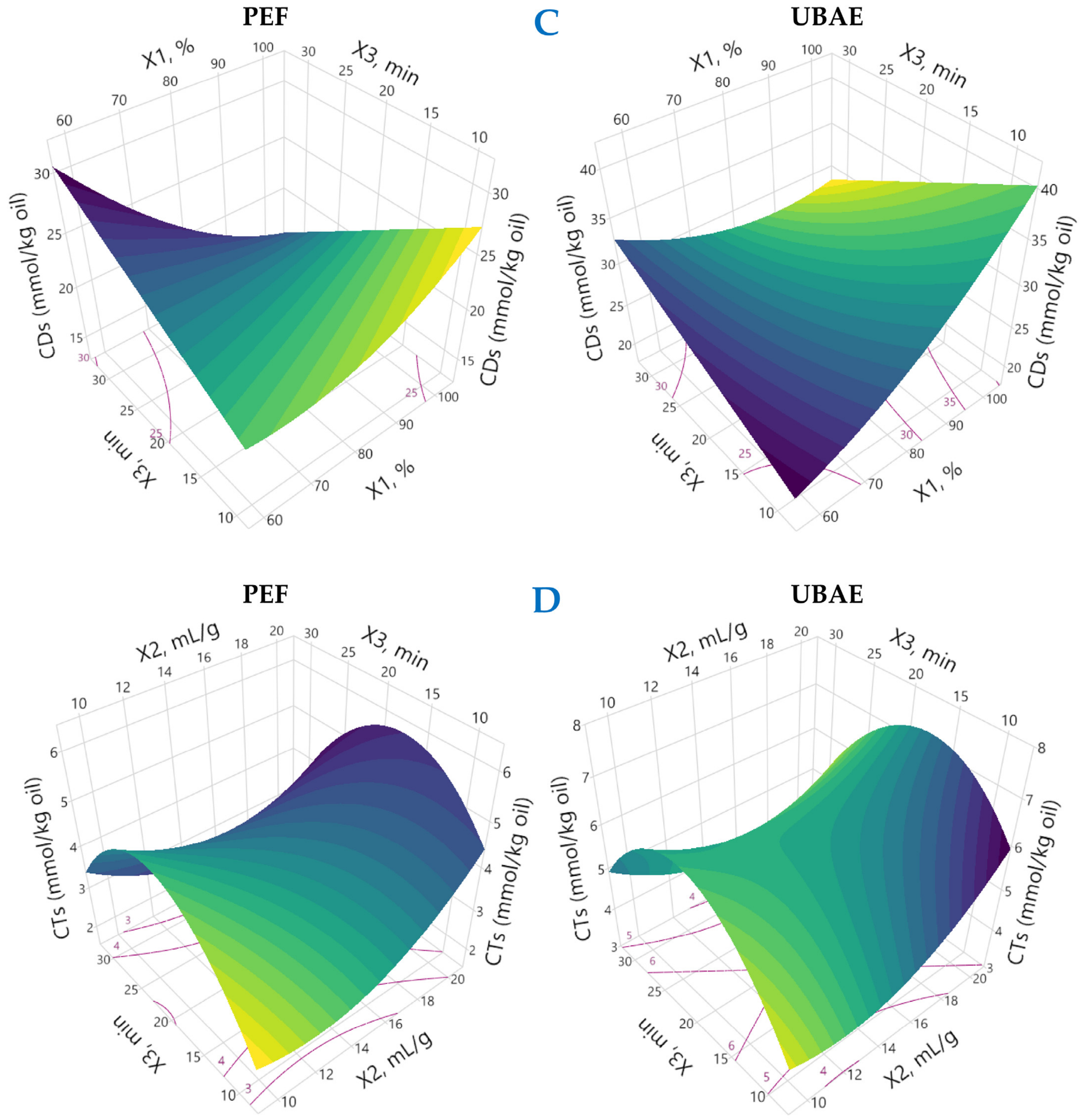

Figure S1. Three-dimensional response surface plots illustrating the effects of extraction time (X_3), liquid-to-solid ratio (X_2), and energy power (X_1) on (**A**) fat yield, (**B**) DPPH radical scavenging activity, (**C**) conjugated dienes (CDs), and (**D**) conjugated trienes (CTs) under PEF and UBAE conditions.
